# Expression of Aurora Kinase A and B in chondrosarcoma and its relationship with the prognosis

**DOI:** 10.1186/1746-1596-7-84

**Published:** 2012-07-18

**Authors:** Xiaohui Liang, Danying Wang, Yan Wang, Zhiqiang Zhou, Juan Zhang, Jinsong Li

**Affiliations:** 1 Department of Pathology, Qilu Hospital of Shandong University, Jinan 250012, China; 2 Department of Admission office, Qilu Hospital of Shandong University, Jinan 250012, China

**Keywords:** Aurora kinase A, Aurora kinase B, Chondrosarcoma

## Abstract

**Purpose:**

To investigate the expression of Aurora Kinase A and B in patients with chondrosarcoma and consider it as a prognostic marker and molecular target of therapy.

**Methods:**

To evaluate the relationship of the Aurora Kinase A and B and the clinical pathological parameters and prognosis of chondrosarcoma. 72 case chondrosarcoma and 42 case chondroma were performed immunohistochemistry on the tissue microarray paraffin sections. The survival time of patients was followed-up.

**Results:**

The expression of Aurora Kinase A and B in chondrosarcoma was significantly higher than that in chondroma (*p<0.01*). There were differences about the expression of Aurora Kinase A and B in chondrosarcoma between the recurrence group and the non-recurrence group, metastatic group and non-metastatic group (*p<0.05*), but not age and gender (*p>0.05*). The expression of Aurora Kinase A and B were significantly lower in group low grade conventional chondrosarcoma than that in groups medium and high grade conventional chondrosarcoma (*p<0.01*). The expression of Aurora Kinase A and B in chondrosarcoma showed a positive correlation (*p<0.01*). According to the Kaplan Meier analysis and multivariate Cox regression analysis, the survival rate was significantly different between the patients with positive Aurora Kinase A and the patients with negative expression (*p<0.05*) and Aurora Kinase A expression was an independent risk marker of survival(*HR=11.263*, *95%CI: 2.317–54.748, P=0.003*).

**Conclusion:**

Both the Aurora Kinase A and B might involve in the oncogenic, invasive and metastatic process of chondrosarcoma; however, the mechanism is still unclear. The Aurora Kinase A and B could be used as a new prognostic marker and molecular therapeutic target for chondrosarcoma.

**Virtual Slide:**

The virtual slide(s) for this article can be found here: http://www.diagnosticpathology.diagnomx.eu/vs/9101494267377096.

## Introduction

Chondrosarcoma is the second most common malignant bone tumors following the osteosarcoma in China [1], in which the tumor cells can generate a lot of bone matrix. To some extent, the chondrosarcoma is not sensitive to chemotherapy and radiation therapy, due to its characteristic of increase of extracellular matrix, decrease of divided cells vessels. To date, the dominant clinical treatment for chondrosarcoma is surgical resection; however, the recurrence rate is too high (20%). Therefore, there is an urgent need of a new therapy for patients with chondrosarcoma, especially for patients with recurrence.

Aurora Kinase family is a newly discovered serine and threonine kinase family, which regulates the function of centriole and microtubule, and plays an important role in maintaining the normal mitosis and cell cycle. However, the disordered expression of Aurora Kinase promoted the oncogenesis. The Aurora Kinase family consists of 3 members in mammal: Aurora Kinase A, B and C. In recent study, it showed that tumorigenesis appeared by transfection of the Aurora Kinase A gene into 3T3 cells in nude mouse [2], and the Aurora Kinase A gene had been identified as a new oncogene. Aurora Kinase A gene was highly expressed in various malignant tumors, such as oophoroma, colorectal cancer and acute myeloid leukemia [3-5]. Aurora Kinase B was also highly expressed in many tumor tissues, including oral cancer, the non-small-cell lung carcinoma and hepatocellular carcinoma [6-8], etc. Furthermore, it has been showed that the activity inhibitors of Aurora Kinase could inhibit the growth of cancer cells, and some inhibitors have been applied in the pre-clinical experimental stage [9,10].

Whether the Aurora Kinase A and B were expressed in the chondrosarcoma is still unknown, in this study, we investigated the expression of the Aurora Kinase A and B in the chondrosarcoma tissues by immunohistochemistry and analyzed its clinical significance; and we also followed-up the patients.

## Materials and methods

### Tissue samples

72 chondrosarcoma samples and 42 chondroma samples were randomly chosen from the Department of Pathology in Qilu Hospital of Shandong University from January 2005 to June 2011, all samples were fixed with 10% formaldehyde and embedded with paraffin. Meanwhile, follow-up survey was conducted by telephone. The clinical parameters included the age, gender, pathological type and the status of recurrence, metastasis and invasion, as shown in Table 1. Informed consent was obtained from all of the patients.

**Table 1:** Clinicopathological parameters of the involved chondrosarcoma cases

**Clinicopathological parameters**	**N(case)**
Age(year)	
<50	49
≥50	23
Gender	
male	35
female	37
Recurrence	
positive	20
negative	52
Metastatic	
positive	24
negative	48
Typing	
conventional type	40
I	26
II	6
III	8
other type	32
Follow-up	
average median survival time	46(month)
average follow-up interval	39.4(month)

### Tissue microarray (TMA)

Firstly, all samples were performed hematoxylin and eosin staining (HE), then two fields were chosen from all HE slides and the corresponding spots were marked on the surface of the paraffin block. Next the marked areas were punched out and placed into the recipient block side by side with the tissue microarray punching instrument. Each tissue point was 2 mm in diameter and assigned with a unique tissue microarray location number, which was linked to a database containing other clinicopathologic data.

### Immunohistochemistry assay

The streptavidin-peroxidase-biotin (SP) immunohistochemical method was used to study the expression of Aurora Kinase A and B in the tissue microarrays. The paraffin specimens were cut into 4 μm sections and stayed at 60°C for 60 min. Then the sections were dewaxed and rehydrated. Next, antigen retrieval was achieved by microwave using EDTA buffer for 2.5 min and then cooled at room temperature for 30 min. After washing with PBS for 3 times, the endogenous peroxidase was blocked with 3% hydrogen peroxide, followed by incubation with goat serum to block nonspecific binding. Then rabbit anti-human polyclonal Aurora-A antibody (1:800; ABGENT,American) and rabbit anti-human polyclonal Aurora-B antibody (1:1000; ABGENT,American) were incubated with the sections overnight at 4°C. After washing, the tissue sections were treated with biotinylated anti-rabbit secondary antibody (Zhongshan Biotechnology Company, Beijing, China) for 30 min; then incubated with streptavidin-horseradish peroxidase complex for 20 min. At last, the sections were developed with diaminobenzidine (DAB) and counterstained with hematoxylin. For negative controls, the Aurora-A antibodies and the Aurora-B antibodies were replaced by PBS.

### Evaluation of immunohistochemical staining

3 visual fields (400×) were randomly chosen and cells were counted with Image-Pro Plus (Media Cybernetics, Bethesda, MD). The Aurora Kinase A is expressed in cytoplasm and the Aurora Kinase B is expressed in both cytoplasm and nucleus. Grade and score by the intensity of staining: 0 point for colorless, 1 point for light yellow, 2 points for claybank and 3 points for brown; then grade by the ratio of positive cell: 0 point for no positive staining cells, 1 point for positive ratio ≤10%, 2 points for 11%–50%, 3 points for 51%–75% and 4 points for>75%. At last multiply the 2 score system: ≤3 points for negative, >3 points for positive. The slides were reviewed and scored independently by two observers who blinded to the patients’ information.

### Statistical analysis

Data were analyzed by SPSS17.0 statistical analysis software. A *P<0.05* was considered statistically significant.

## Results

### Expression of Aurora Kinase A and B in chondrosarcoma and chondroma

**Figure 1:**
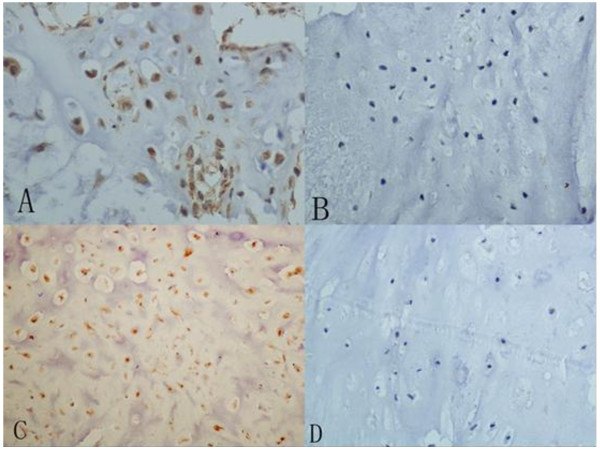
**Expression of Aurora Kinase A and B testing by Immunohistochemistry method (×400).A**. the positive expression of Aurora Kinase A in chondrosarcoma **B**. the negative expression of Aurora Kinase A in chondroma **C**. the positive expression of Aurora Kinase B in chondrosarcoma **D**. the negative expression of Aurora Kinase B in chondroma.

**Figure 2:**
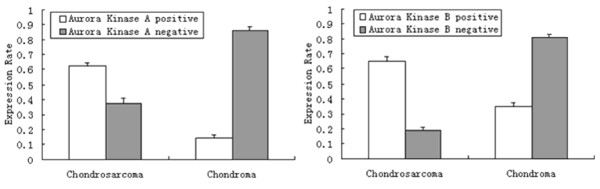
Comparison of the expression rate of Aurora Kinase A and B in chondrosarcoma and chondroma.

The Aurora Kinase A protein was observed mainly in the cytoplasm (Figure 1A), and it was positive in 45 of the 72 patients (62.5%) with chondrosarcoma and in 6 of the 42 cases (14.3%) with chondroma (Table 2). The positive ratio of Aurora Kinase A was significantly higher in chondrosarcoma than that in chondroma tissues (*p<0.01*) (Figure 2). The Aurora Kinase B protein was detected in both the nucleus and the cytoplasm (Figure 1C). The positive ratio of Aurora Kinase B was 65.3% (47/72) in the chondrosarcoma and 19% (8/42) in the chondroma tissues (Table 2). The positive ratio of Aurora Kinase B was significantly higher in the chondrosarcoma than that in chondroma tissues (*p<0.01*) (Figure 2).

**Table 2:** Expression of Aurora Kinase A and B in the tissue of chondrosarcoma and chondroma

**Group**	**Chondrosarcoma**	**Chondroma**	**χ**^**2**^**value/P value**
Aurora Kinase A positive	45	6	24.942/0.000
Aurora KinaseA negative	27	36	
Aurora KinaseB positive	47	8	22.705/0.000
Aurora KinaseB negative	25	34	

### Expression of Aurora Kinase A and B and chondrosarcoma

The ratio of positive expression of the Aurora Kinase A was 33.3%and 92.9% inIandII-III stage of the conventional chondrosarcoma, respectively *(p<0.01*); the more malignant tumor, the higher expression of the Aurora Kinase A. The ratio of positive expression was 90% and 51.9% in the group with and without recurrence, respectively (*p<0.05*). The ratio of positive expression was 83.3% and 52.1% in the metastatic and non-metastatic group, respectively (*p<0.05*). However, the expression of Aurora Kinase A was not correlated with age, gender *(p>0.05*) (Table 3).

**Table 3:** Association of Aurora Kinase A,B expression with the clinicopathological features of the chondrosarcoma

**Clinicopathological parameters**	**N**	**Aurora**	**Kinase A**	***χ***^**2**^**value**	**P value**	**Aurora**	**Kinase B**	***χ***^**2**^**value**	**P value**
		**positive**	**negative**			**positive**	**negative**		
Age(year)									
<50	49	32	17	0.515	0.473	29	20	2.513	0.113
≥50	23	13	10			18	5		
Gender									
male	35	20	15	0.83	0.361	22	13	0.176	0.675
female	37	25	12			25	12		
Recurrence									
positive	20	18	2	7.385*	0.007	17	3	3.624*	0.047
negative	52	27	25			30	22		
Metastatic									
positive	24	20	4	5.4*	0.020	21	3	6.442*	0.011
negative	48	25	23			26	22		
Conventional type									
I	27	9	18	10.85*	0.001	9	18	14.04*	0.000
II-III	14	13	1			14	0		

*using continuity correction Chi-Square.

The ratio of positive expression of Aurora Kinase B protein in common chondrosarcoma groupIandII-III was 33.3% and 100% respectively (*p<0.01*); which means the more malignant tumor, the higher expression of the Aurora Kinase B. The ratio of positive expression was 85% and 57.7% in the recurrence group and non-recurrence group, respectively (*p<0.05*). The expression of Aurora Kinase B was not correlated with age, gender too. The expression of Aurora Kinase A was positively correlated with the expression of Aurora Kinase B (*p<0.01*) (Table. 4). The Aurora Kinase A and B were negative in negative controls.

**Table 4:** Correlation of the expression of Aurora Kinase A and B in chondrosarcoma

**Aurora Kinase A**	**Aurora Kinase B**		***χ***^**2**^**value**	**P value**
	**+**	**-**		
+	40	5	28.277	0.000
-	7	19		

### Survival analysis

**Figure 3:**
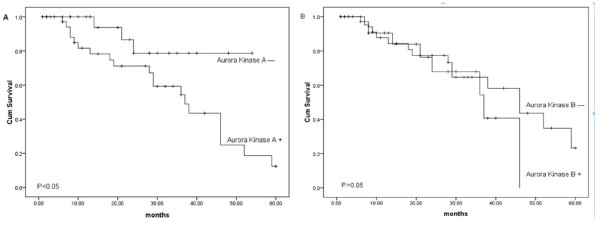
**The Kaplan-Meier survival analysis of 72 patients with chondrosarcoma.A**: The analysis with Kaplan-Meier method clearly showed that patients with chondrosarcoma negative for Aurora Kinase A had higher overall survival rates than those positive for Aurora Kinase A. **B**: The expression of Aurora Kinase B shows no significant relationship with the overall survival rates of the patients with chondrosarcoma

The survival time of the patients was followed-up and analyzed with the Kaplan-Meier survival analysis and multivariate Cox regression analysis method. The average follow-up interval was 39.4 months and the average median survival time was 46 months. The results of Kaplan-Meier survival analysis showed that chondrosarcoma patients who had a positively expressed Aurora Kinase A had a relatively poor prognosis (*p<0.05*) (Figure 3) which indicates that the Aurora Kinase A might be a prognostic marker for the chondrosarcoma. The results of multivariate Cox regression analysis showed that Aurora Kinase A was an independent risk marker of overall survival(*HR=11.263, 95%CI: 2.317–54.748, P=0.003*) (Table 5), However, there was no significant relationship between Aurora Kinase B and the survival time of the patients with chondrosarcoma (*p>0.05*). Aurora Kinase B was not an independent risk marker of overall survival (*HR=0.246, 95%CI: 0.070–0.869, P=0.029*).

**Table 5:** Multivariate Cox regression analysis of Aurora Kinase A and B and clinicopathological features

	**Category**	**Hazard Ratio**	**P-value**	**95.0%CI for HR**	
				**Lower**	**Upper**
Gender	Male/Female	1.033	0.945	0.409	2.609
Age	<50/≥50	2.370	2.370	0.764	7.351
Recurrence	No/Yes	0.464	0.120	0.176	1.221
Metastatic	No/Yes	1.088	0.879	0.365	3.249
Aurora Kinase A	+/−	11.263	0.003	2.317	54.748
Aurora Kinase B	+/−	0.246	0.029	0.070	0.869

## Discussion

Tumor is a type of general, systematic and step-by-step developing disease involving lots of factors; it is caused by the mutation of the oncogenes and disorders of some genes [11]. Chondrosarcoma derived from the chondrocytes, which is a primary malignant tumor, which consists of the tumorous chondrocytes and cartilage matrix. The prevalence of chondrosarcoma was approximately 20% of all the primary bone malignant tumors [12]. At present, the research on molecular targeted therapy for malignant tumors is popular all over the world. The molecular targeted therapy mainly corrects the abnormal signaling pathways of the tumor, with the characteristics of high selectivity, hypotoxicity and high therapeutic index [11]. The abnormal expression of Aurora Kinase family is closely associated with the development of tumor, because of the crucial role of Aurora Kinase family plays in the mitosis and cell cycle. In this report, we investigated the expression of Aurora Kinase A and B in chondrosarcoma and provided a strong support for the development of the new drugs of molecular targeted therapy for chondrosarcoma.

Aurora Kinase A is a member of Aurora Kinase family which has been considered as a crucial factor to regulate mitosis. It participates in the maturation and segregation of centriole, the assembly and stabilization of spindle, the condensation of chromosome and the converging and stabilization of the median plate, etc. However, its overexpression can result in the augmentation of centriole, the formation of multiploid and the loss of p53. Through studying the expression profile of Aurora Kinase A in various normal tissues, we found that Aurora Kinase A mRNA only had a relative low expression level in thymus, but was barely detected in other tissues, such as the brain, lung, intestine, spleen and testis. In various malignant tumors Aurora Kinase A had been found overexpression. Marco et al. reported that the expression of Aurora Kinase A was up-regulated in hepatoma especially in the type of poorly differentiated tissues [13]. Rudolf et al. reported that the expression of Aurora Kinase A mRNA was up-regulated in the head and neck carcinoma [14]. Therefore, it could be considered as a prognostic factor for malignant tumors.

Our results showed that the expression of Aurora Kinase A was very low in chondroma tissues, while it was strongly expressed in chondrosarcoma; which indicated that Aurora Kinase A played an important role in the initial and developmental stage of the chondrosarcoma. The results also showed that the expression of Aurora Kinase A was significant different between the recurrence group and the non-recurrence group, the metastatic group and non-metastatic group, suggesting that the high expression of Aurora kinase A is associated with oncogenesis and the grade of the malignancy and differentiation of chondrosarcoma. However, there was no correlation of the expression of Aurora Kinase A and the age, gender, the site and size of the tumor. Combination of immunohistochemisty and follow-up data we can see that there was significant difference of the overall survival rate between the Aurora Kinase A positively expressed group and the negatively expressed group.

In addition, it has been reported that the high expression of Aurora Kinase A in esophageal squamous cell carcinoma could significantly promote the proliferation of tumor cells and increase the resistance to apoptosis induced by DDP and ultraviolet radiation [15]. Takayuki et al. reported that Aurora Kinase inhibitor ZM447439 could inhibit the growth of leukemia cells [5]. Thereforethe inhibition of the activity of Aurora Kinase A could lead to the tumor cell apoptosis instead of malignant proliferation. Accordingly, Aurora Kinase A is expected to become the new target for chondrosarcoma. In the further study, we will further conduct related experiments to identify whether the Aurora Kinase A inhibitor can be used as the molecular target treatment for chondrosarcoma.

The Aurora Kinase B is the component of the chromosome passenger protein complex which participate in the chromosome condensation, spindle assembly, segregation of sister chromatids and cytocinesis. Recent studies reported that Aurora Kinase B expressed abnormally in various malignant tumors, such as breast cancer [16] and acute lymphatic leukemia [17]. Our results showed that in chondroma tissues the expression of Aurora Kinase B was barely detected, however, most of the chondrosarcoma tissue cells expressed positively, even 100% positive in stage III.It indicated that Aurora Kinase B played an important role in the process of the oncogenesis and development of chondrosarcoma. Moreover, we found the positive expression of Aurora Kinase B was significant different between the metastatic group and non-metastatic group and the recurrence group and the non-recurrence group, which suggests that Aurora Kinase B impacts on the invasiveness of chondrosarcoma. Ren et al. reported that it might enhance the sensitivity of ovarian cancer cell A2780 against chemotherapy to paclitaxel and increase the apoptosis by application of AURKB siRNA to A2780, indicating that inhibit of Aurora Kinase B could finally restrain the neoplastic growth by inducing apoptosis of tumor cells [18]. A variety of Aurora Kinase inhibitors have been developed, such as hesperetin against Aurora Kinase B, quinazoline against Aurora Kinase A and B simultaneously and vX-680 against Aurora Kinase A, B and C; besides, some inhibitors have been applied in pre-clinical and I,II stage clinical trials [19]. In the future, our group will study whether Aurora Kinase B inhibitors can inhibit the growth and proliferation of chondrosarcoma cells in vitro and in vivo. Dual inhibitors may be a better choice due to the consistent expression of Aurora Kinase A and Aurora Kinase B.

## Conclusion

Our study found that the expression of Aurora Kinase A and B was significantly up-regulated in chondrosarcoma, and the expression of Aurora Kinase A and B was correlated with the recurrence and metastasis of chondrosarcoma. Which demonstrate that Aurora Kinase A and B play an important role in the formation and development of chondrosarcoma. In addition, survival analysis and multivariate Cox regression analysis showed that Aurora Kinase A also affected patients’ outcome. Therefore, Aurora kinase A can be used to predict the prognosis of patients with chondrosarcoma.

## Competing interests

The authors declare that they have no competing interests.

## Authors’ contributions

XL and YW did the immunohistochemical analysis. DW and ZJ reviewed all the pathological slides and made the tissue microarray. ZZ analyzed the data. JL designed the study. All authors read and approved the final manuscript.
